# Optimizing a Multimodal Large Language Model for Ultrasound-Based Thyroid Nodule Malignancy Classification: A Comparative Study of Few-Shot Learning, Prompt Engineering, and Fine-Tuning

**DOI:** 10.3390/diagnostics16121931

**Published:** 2026-06-22

**Authors:** Yu-Hsuan Li, Yu-Cheng Cheng, Chih-Yun Chang, I-Te Lee

**Affiliations:** 1 Department of Computer Science & Information Engineering, National Taiwan University, Taipei 106319, Taiwan; 2 Division of Endocrinology and Metabolism, Department of Internal Medicine, Taichung Veterans General Hospital, Taichung 407219, Taiwan; 3 Department of Digital Medicine, Taichung Veterans General Hospital, Taichung 407219, Taiwan; 4 School of Medicine, National Yang Ming Chiao Tung University, Taipei 112304, Taiwan; 5 School of Medicine, Chung Shan Medical University, Taichung 402306, Taiwan; 6 Department of Post-Baccalaureate Medicine, College of Medicine, National Chung Hsing University, Taichung 402002, Taiwan

**Keywords:** atypia of undetermined significance, multimodal large language model, nodule, thyroid, ultrasound

## Abstract

**Objectives:** Multimodal large language models (MLLMs) have shown potential for medical image classification. We evaluated four optimization strategies in two MLLMs—GPT-4o (gpt-4o-2024-08-06) and Gemini 2.5 Flash-Lite—for ultrasound-based thyroid nodule malignancy classification using two public datasets and a clinical cohort of nodules with atypia of undetermined significance (AUS) cytology. **Methods:** Text prompting, few-shot learning, fine-tuning, and a hybrid strategy combining fine-tuning with few-shot learning were evaluated for each model. Performance was assessed using the Digital Database of Thyroid Images (DDTI; *n* = 80), a 1000-image test subset of TN5000, and an institutional AUS cohort with surgical pathology (*n* = 84). In the AUS cohort, the best-performing strategy was compared with the consensus classification of three endocrinologists and the American Thyroid Association (ATA) ultrasound risk stratification. **Results:** For GPT-4o, the hybrid strategy achieved the highest area under the receiver operating characteristic curve (AUC) in DDTI (0.866), TN5000 (0.689), and the AUS cohort (0.836). In the AUS cohort, its specificity was higher than that of endocrinologist consensus and ATA risk stratification when only high-suspicion nodules were classified as malignant (95.1% vs. 70.7% and 70.7%; *p* = 0.002 and *p* = 0.001, respectively), while sensitivity did not differ significantly (72.1% vs. 74.4% and 79.1%, respectively; both *p* > 0.05). However, the hybrid model misclassified 12 of 43 malignant nodules, corresponding to a false-negative rate of 27.9%. When high- and intermediate-suspicion ATA categories were classified as malignant, ATA sensitivity increased to 83.7% and specificity decreased to 56.1%; the hybrid model had a higher AUC than ATA risk stratification (0.836 vs. 0.749; *p* = 0.017). For Gemini 2.5 Flash-Lite, few-shot learning, fine-tuning, and the hybrid strategy did not improve AUC relative to text prompting in any dataset. **Conclusions:** The hybrid strategy produced the most consistent performance gains for GPT-4o across the three datasets but did not improve Gemini 2.5 Flash-Lite. The optimized GPT-4o model achieved high specificity in the diagnostically challenging AUS cohort, although its false-negative rate limits its use as a stand-alone diagnostic tool. Further validation in larger, prospective multicenter cohorts is required before clinical use.

## 1. Introduction

Thyroid ultrasound is the cornerstone of thyroid nodule evaluation [1]. Several ultrasound risk stratification systems, including the American Thyroid Association (ATA) Risk Stratification System and the Thyroid Imaging Reporting and Data System (TIRADS) [2,3], have been developed and widely adopted to differentiate benign and malignant thyroid nodules [4,5]. While these systems have improved diagnostic consistency and clinical decision-making, challenges persist, especially for nodules with indeterminate cytology such as Bethesda category III, i.e., Atypia of Undetermined Significance (AUS). Ultrasound risk stratification has been reported to be less reliable for this category of nodules, which carry a 15–30% risk of malignancy [3,6,7]. The growing volume of thyroid ultrasound examinations is also increasing the clinician workload. Collectively, these factors highlight the need for tools that offer improved diagnostic accuracy and efficiency.

Deep learning-based computer-aided diagnosis systems have shown strong performance in thyroid ultrasound classification [8]. However, many models depend on large, carefully annotated image datasets and their performance may degrade during external validation, which limits their clinical use [9]. The multimodal large language models (MLLMs) represent a promising approach for medical image interpretation [10]. While MLLMs have shown broad general capability [11], their performance on specific medical image classification tasks remains inconsistent and generally inferior to that of experts [12,13,14,15]. Recent multi-model evaluations across clinical domains have revealed that performance varies considerably between models and tasks, and that structured, expert-anchored assessment is needed before clinical use [16,17]. Previous research suggests that optimization strategies such as prompt engineering, few-shot learning, and fine-tuning can individually improve MLLM performance; however, a systematic evaluation of their comparative efficacy, synergistic potential, and post-optimization generalizability is still lacking [18].

Accordingly, this study aims to evaluate four optimization strategies for MLLMs to classify thyroid nodules on ultrasound: text prompting, few-shot learning, fine-tuning, and a hybrid strategy combining fine-tuning with few-shot learning. We evaluated two contrasting models—GPT-4o, a non-reasoning MLLM from the GPT family, and Gemini 2.5 Flash-Lite, a reasoning model that supports a configurable thinking budget enabling explicit chain-of-thought reasoning—to test whether the same optimization strategies transfer across model families. We assessed these four optimization strategies using the public Digital Database of Thyroid Images (DDTI) and a 1000-image test split of the TN5000 dataset [19], which has a markedly higher malignancy prevalence. We assessed clinical relevance in an AUS cohort with surgically confirmed histopathology. Finally, we benchmarked the best-performing optimization strategy against the experienced endocrinologists’ consensus and the ATA ultrasound risk stratification.

## 2. Materials and Methods

### 2.1. Study Design

**Figure 1:**
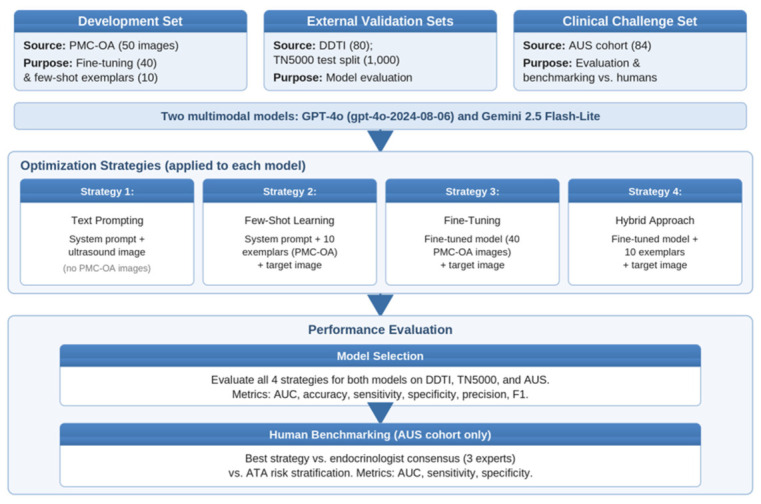
Study design and evaluation workflow. Overview of the development dataset from PubMed Central Open Access (PMC-OA, access on 23 January 2025) used for fine-tuning and few-shot exemplars, external validation on the Digital Database of Thyroid Images (DDTI) and the 1000-image test split of TN5000, and clinical challenge evaluation on the AUS cohort. Four strategies were evaluated for both GPT-4o and Gemini 2.5 Flash-Lite: text prompting, few-shot learning, fine-tuning, and a hybrid approach (fine-tuned model plus few-shot exemplars at inference). The best-performing strategy was benchmarked against endocrinologist consensus and American Thyroid Association (ATA) risk stratification on the Atypia of Undetermined Significance (AUS) cohort.

We conducted a comparative evaluation of four optimization strategies for thyroid nodule classification on ultrasound images: (1) text prompting, (2) few-shot learning, (3) fine-tuning, and (4) a hybrid approach combining fine-tuning with few-shot learning. Each strategy was applied to two MLLMs: GPT-4o (gpt-4o-2024-08-06, OpenAI, San Francisco, CA, USA) and Gemini 2.5 Flash-Lite (Google), a reasoning model evaluated with a fixed reasoning budget (thinkingBudget = 512). The Gemini reasoning budget was fixed at thinkingBudget = 512 tokens, a moderate value within the supported range that allowed chain-of-thought reasoning while controlling latency and cost. In preliminary testing on a small held-out subset, this budget produced stable, well-formed, structured outputs, and larger budgets did not change the binary classifications; the budget was therefore held constant across all Gemini experiments to keep the comparison consistent. Model performance was validated on three independent ultrasound datasets with differing malignancy prevalence. The best-performing strategy was subsequently benchmarked against experienced endocrinologists using a diagnostically challenging dataset of nodules with AUS (Figure 1).

### 2.2. Datasets

#### 2.2.1. Evaluation Datasets

- **DDTI:** For model evaluation, we used a curated subset of the publicly available DDTI “https://www.kaggle.com/datasets/eiraoi/thyroidultrasound (accessed on 20 January 2025)” [20]. The reference label was the benign/malignant designation provided in the original dataset. Two board-certified endocrinologists independently selected images from the original 347 images based on predefined quality criteria: (i) grayscale B-mode thyroid ultrasound with adequate resolution; (ii) a clearly visible dominant nodule with margins and internal echotexture not obscured by shadowing, motion, or major artifacts; (iii) minimal burned-in annotations or calipers that could conceal key features; and (iv) a clear benign/malignant label provided by the dataset. Of the 347 original images, 125 were excluded as duplicate views of the same nodule, 78 for shadowing, motion, or major artifacts obscuring the nodule, 13 for burned-in calipers or annotations concealing key features, and 51 for the absence of a clear benign/malignant label. Exclusions targeted image quality and labeling adequacy rather than diagnostic difficulty. Only images meeting all criteria and with reviewer consensus were included, yielding 80 images (28 malignant, 52 benign).
- **TN5000 Test Split:** To evaluate generalizability under a higher malignancy prevalence, we used the publicly described 1000-image test split of the TN5000 thyroid ultrasound dataset (731 malignant, 269 benign; malignancy prevalence 73.1%). TN5000 is a large grayscale B-mode thyroid ultrasound collection in which benign and malignant labels were assigned based on fine-needle aspiration and surgical pathology. The standard 1000-image test split was used as released, without additional curation, to provide an independent external test set whose case mix differs markedly from DDTI and the AUS cohort. Malignant nodules were treated as the positive class [19].
- **AUS Dataset:** To assess performance on a clinically challenging cohort, a private dataset of 84 thyroid ultrasound images was retrospectively collected from Taichung Veterans General Hospital. These images were from patients with at least two AUS cytology results on fine-needle aspiration (FNA) who subsequently underwent surgical resection for definitive histopathological diagnosis, yielding 43 malignant and 41 benign nodules. An experienced endocrinologist confirmed the image quality prior to inclusion. This dataset was also used for the human expert comparator arm. The endocrinologist consensus and ATA comparators were applied only to the AUS cohort with definitive surgical histopathology; this represents the targeted clinical challenge. The DDTI and TN5000 datasets were used for external technical validation of the optimization strategies.

#### 2.2.2. Development Dataset (For Fine-Tuning and Few-Shot Examples)

- **PubMed Central Open Access (PMC-OA) Dataset:** A distinct set of 60 de-identified thyroid ultrasound images was curated from the PMC-OA repository (available at https://huggingface.co/datasets/axiong/pmc_oa, access on 23 January 2025) [21] to serve as the source for fine-tuning data and few-shot learning examples. Images were selected based on clear nodule depiction and caption-derived diagnostic information, validated by two endocrinologists with 7 and 15 years of experience in thyroid ultrasonography. These were the same two board-certified endocrinologists who curated the DDTI subset in Section 2.2.1; the two datasets were annotated in separate sessions to limit fatigue. These endocrinologists provided structured annotations based on ATA guidelines [1], including binary benign/malignant labels. The development set was divided into two independent subsets: a 50-image fine-tuning set (40 training and 10 validation images) and a separate 10-image few-shot set. Across the fine-tuning set, there were 21 malignant and 29 benign images (training: 26 benign, 14 malignant; validation: 3 benign, 7 malignant), and the 10 few-shot exemplars comprised 5 malignant and 5 benign images. The same 40-image training set and 10-image validation set were used to fine-tune both GPT-4o and Gemini 2.5 Flash-Lite. The few-shot, fine-tuning training, and fine-tuning validation subsets were mutually exclusive, and all were independent of the DDTI, TN5000, and AUS evaluation sets.

#### 2.2.3. Optimization Strategies

GPT-4o experiments used the OpenAI API between 15 November 2024 and 3 December 2024, with the model identifier gpt-4o-2024-08-06. Gemini experiments used Gemini 2.5 Flash-Lite with thinkingBudget = 512. The same system prompt and output schema were used across the four strategies and both models (Supplementary Materials). The step-by-step inference workflow was as follows. (i) De-identification and formatting: All ultrasound images were de-identified before analysis and provided to the API in a standard raster format without additional preprocessing that could alter sonographic appearance. (ii) Model call: The system prompt (and, when applicable, few-shot exemplars) plus the query image were submitted via the API. (iii) Output parsing: Responses were programmatically parsed according to the predefined JSON schema. (iv) Prediction extraction: The primary predicted label (benign vs. malignant) was extracted and used for confusion matrix-based metrics. The ATA risk pattern, when provided, was treated as an ordered score for ROC analysis.

- **Text Prompting:** The prompt directed each model to perform a structured analysis based on key elements of the ATA ultrasound risk stratification guidelines [1]. The model was instructed to (i) describe key sonographic features (composition, echogenicity, shape, margins, and echogenic foci), (ii) map these features to ATA risk patterns, and (iii) output a final binary classification (benign vs. malignant) along with the corresponding ATA suspicion category (high/intermediate/low/very low/benign). During text prompting, no images from the PMC-OA development set were used; it relied solely on the ATA-anchored system prompt. Requesting structured ATA features provides an ordered suspicion score for ROC analysis and renders the model’s reasoning interpretable, which is relevant for clinical transparency even though the primary endpoint is binary classification.
- **Few-Shot Learning:** For few-shot learning, we used 10 examples (5 malignant, 5 benign) from the PMC-OA development dataset, accompanied by their labels and concise feature descriptions. These 10 examples were selected by consensus of two endocrinologists as images that (i) displayed textbook ATA sonographic features for their category, (ii) had unambiguous benign/malignant labels supported by the source caption, and (iii) were of high quality without obscuring artifacts. Images with mixed or borderline features were not used as exemplars, so that each example presented a clear prototype of its class. The two endocrinologists reviewed each candidate image independently; because selection was consensus-based rather than independent parallel scoring, a formal inter-rater agreement coefficient (e.g., Cohen’s kappa) was not calculated. The few infrequent disagreements, which concerned borderline images, were resolved by discussion, and any image on which consensus could not be reached was excluded rather than assigned a forced label.
- **Fine-Tuning:** From the PMC-OA development dataset, 40 images (distinct from the few-shot exemplars) were allocated for fine-tuning, with a further 10 images held out for validation. These strategies use different numbers of labeled development images by design—text prompting uses none, few-shot learning uses 10 in-context exemplars, and fine-tuning uses 40 training images and 10 images for validation—so the comparison reflects each strategy as it is realistically deployed under a single fixed development budget rather than a controlled equal-N comparison. Images were paired with expert annotations in JSONL format. For GPT-4o, fine-tuning was executed via the OpenAI API using the default learning rate multiplier of 1.0 with 3 epochs and a batch size of 1, chosen to limit overfitting risk with the small training set based on preliminary testing. For Gemini 2.5 Flash-Lite, supervised tuning was carried out using the same 40 annotated images. The base model Gemini-2.5 Flash-Lite was used, with 3 epochs, a learning-rate multiplier of 0.5, and an ADAPTER_SIZE_ONE adapter size, on a training set of 40 images (26 benign, 14 malignant) and a validation set of 10 images (3 benign, 7 malignant). The same 40-image training set and 10-image validation set were used for both GPT-4o and Gemini 2.5 Flash-Lite. The validation subset was weighted toward malignant cases relative to the training set and was not rebalanced owing to the limited size of the development set. The OpenAI fine-tuning interface exposes only step-level training loss and periodic validation loss for GPT-4o, which are shown together with the Gemini 2.5 Flash-Lite training and validation loss in Supplementary Figure S1. Training and validation loss curves were monitored for convergence and signs of overfitting, and JSONL files underwent programmatic validation for structural integrity.

### 2.3. Human Expert Comparator

Three board-certified endocrinologists with 15, 7, and 5 years of post-fellowship experience in thyroid ultrasonography, respectively, independently evaluated the AUS dataset. Each clinician independently reviewed the anonymized ultrasound images, blinded to model outputs and to each other’s initial assessments, and provided a benign/malignant classification. No clinical data beyond the images were provided. The consensus diagnosis for the human expert group was determined by majority agreement (≥2 concordant reads).

### 2.4. ATA Guideline Comparator

To provide a clinical guideline benchmark, the ATA ultrasound risk stratification system was applied to the AUS dataset. Two board-certified endocrinologists independently assigned an ATA risk category to each nodule based on its sonographic features, according to the 2015 ATA guidelines. The 2015 ATA system assigns each nodule to one of five sonographic patterns—high suspicion (estimated malignancy risk 70–90%), intermediate (10–20%), low (5–10%), very low (<3%), or benign (<1%)—based on composition, echogenicity, shape, margin, and echogenic foci. For the primary binary classification analysis, nodules classified as “high suspicion” were considered positive for malignancy, while all other categories were considered benign. Because intermediate-suspicion nodules can also harbor malignancy, we also evaluated a less conservative threshold in which both high and intermediate suspicion were classified as malignant. Any diagnostic discrepancies were resolved by consensus.

### 2.5. Statistical Analysis

Diagnostic performance was quantified using the area under the receiver operating characteristic curve (AUC), accuracy, sensitivity, specificity, precision (positive predictive value), and F1-score. For AUCs, 95% confidence intervals (CIs) were calculated using non-parametric bootstrapping (1000 iterations with replacement); sensitivity, specificity, and accuracy CIs were Wilson intervals; and precision and F1 CIs were obtained by bootstrap resampling. Paired sensitivities, specificities, and accuracies were compared with the mid-*p* version of McNemar’s test, which provides better control of Type-I errors than the exact conditional test while maintaining power for small numbers of discordant pairs [22]. Differences in AUC were assessed with DeLong’s method (two-sided α = 0.05).

For each dataset, model, and optimization strategy, we constructed 2 × 2 confusion matrices using the relevant reference standard. True positives (TPs) were malignant nodules correctly classified as malignant; true negatives (TNs) were benign nodules correctly classified as benign; false positives (FPs) were benign nodules classified as malignant; and false negatives (FNs) were malignant nodules classified as benign. Confusion matrices were visualized to characterize clinically relevant error patterns. A post hoc bootstrap-based power analysis (1000 simulations) indicated that the study had >80% power to detect an absolute difference in AUC (ΔAUC) of ≥0.10 between the optimized GPT-4o model and the human expert group on the AUS dataset, at a two-sided α of 0.05. Statistical analyses were performed using R software (version 4.4; R Foundation for Statistical Computing, Vienna, Austria) with the pROC (v1.18.5) and tidyverse (v1.3.2) packages.

## 3. Results

### 3.1. Cohort Characteristics

Three thyroid ultrasound image datasets were used to evaluate the models, and a fourth was reserved for development (Figure 1). The DDTI evaluation set comprised 80 de-identified images, including 28 (35.0%) malignant and 52 (65.0%) benign nodules. The TN5000 1000-image test split comprised images with a higher malignancy prevalence, including 731 (73.1%) malignant and 269 (26.9%) benign nodules. The AUS evaluation set, collected retrospectively from a single medical center, included 84 images with surgically confirmed pathology, including 43 (51.2%) malignant and 41 (48.8%) benign nodules, while the PMC-OA development set, reserved exclusively for fine-tuning and few-shot learning examples, contained 60 images—an independent 50-image fine-tuning set (21 malignant, 29 benign; 40 training and 10 validation) and a separate 10-image few-shot set (5 malignant, 5 benign)—annotated by experts (Table 1).

### 3.2. Diagnostic Performance on the DDTI Evaluation Set

**Figure 2:**
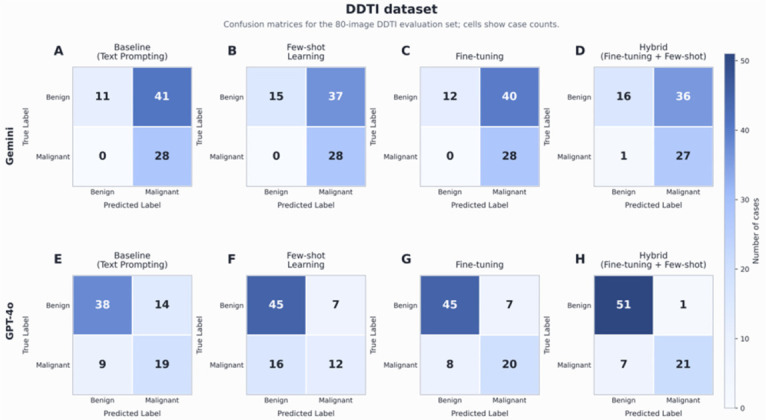
Confusion matrices on the DDTI evaluation dataset. Matrices are shown for Gemini 2.5 Flash-Lite (**A**–**D**) and GPT-4o (**E**–**H**) under each strategy: (**A**,**E**) text prompting, (**B**,**F**) few-shot learning, (**C**,**G**) fine-tuning, and (**D**,**H**) hybrid (fine-tuning + few-shot). Cells show case counts; color intensity encodes count. DDTI was used for external technical validation and was not read by human experts.

The performance of GPT-4o for the four optimization strategies on the DDTI evaluation set is summarized in Table 2, with the corresponding confusion matrices shown in Figure 2E–H. The text prompting strategy achieved an AUC of 0.704 (95% CI: 0.594–0.807), with a sensitivity of 67.9% and specificity of 73.1% (TN/FP/FN/TP = 38/14/9/19; Figure 2E).

Few-shot learning yielded an AUC of 0.647 (95% CI: 0.543–0.750), a nonsignificant change relative to text prompting (ΔAUC −8.1%; *p* = 0.281). Sensitivity decreased to 42.9% with a specificity of 86.5%, consistent with a shift toward fewer false positives but more false negatives (TN/FP/FN/TP = 45/7/16/12; Figure 2F).

Fine-tuning improved discrimination (AUC 0.791, 95% CI: 0.690–0.885), corresponding to a relative AUC increase of 12.4% versus text prompting (*p* = 0.183) and a significant increase versus few-shot learning (ΔAUC +22.3%; *p* = 0.012). The fine-tuned model showed TN/FP/FN/TP = 45/7/8/20 (Figure 2G), with fewer false negatives than few-shot learning.

The hybrid strategy (fine-tuning plus few-shot learning at inference) achieved the highest performance (AUC 0.866, 95% CI: 0.774–0.946), significantly outperforming text prompting (ΔAUC +23.0%; *p* = 0.015) and few-shot learning (ΔAUC +33.9%; *p* < 0.001). This improvement was driven mainly by a marked reduction in false positives while maintaining sensitivity (TN/FP/FN/TP = 51/1/7/21; Figure 2H), giving an accuracy of 90.0%, precision of 95.5%, and F1 of 0.840.

In contrast, for Gemini 2.5 Flash-Lite, none of the optimization strategies improved AUC over text prompting on DDTI (Table 3; Figure 2A–D). Text prompting gave the highest Gemini AUC (0.770), with few-shot learning, fine-tuning, and the hybrid strategy leading to nonsignificant decreases (ΔAUC −8.8% to −2.1%; all *p* > 0.05).

### 3.3. Diagnostic Performance on the TN5000 Test Split

**Figure 3:**
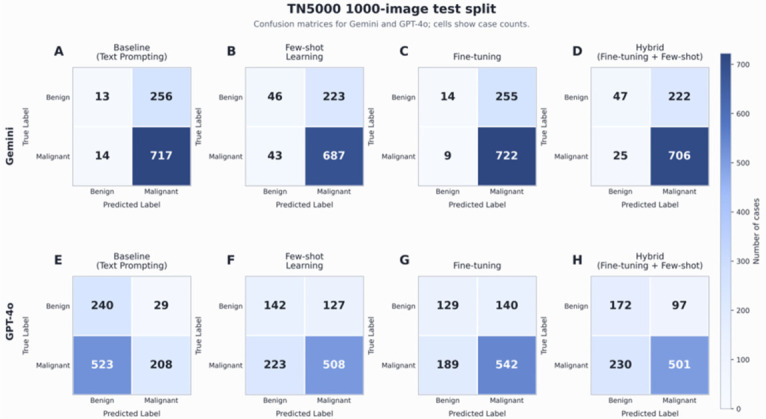
Confusion matrices on the TN5000 1000-image test split. Matrices are shown for Gemini 2.5 Flash-Lite (**A**–**D**) and GPT-4o (**E**–**H**) under each strategy: (**A**,**E**) text prompting, (**B**,**F**) few-shot learning, (**C**,**G**) fine-tuning, and (**D**,**H**) hybrid (fine-tuning + few-shot). Cells show case counts; color intensity encodes count.

The performance of GPT-4o on the higher-prevalence TN5000 test split is summarized in Table 4, with the confusion matrices presented in Figure 3E–H. Text prompting achieved an AUC of 0.573 (95% CI: 0.536–0.607) with high specificity (89.2%) but low sensitivity (28.5%), reflecting a benign-leaning operating point in a predominantly malignant cohort (TN/FP/FN/TP = 240/29/523/208; Figure 3E).

Few-shot learning (AUC 0.613; +7.0%, *p* = 0.076) and fine-tuning (AUC 0.615; +7.3%, *p* = 0.092) increased sensitivity to 69.5% and 74.1%, respectively (TN/FP/FN/TP = 142/127/223/508 and 129/140/189/542; Figure 3F,G).

The hybrid strategy again achieved the highest AUC (0.689, 95% CI: 0.653–0.724), significantly exceeding text prompting (ΔAUC +20.2%; *p* < 0.001), few-shot learning (+12.4%; *p* < 0.001), and fine-tuning (+12.0%; *p* < 0.001), with a more balanced sensitivity (68.5%) and specificity (63.9%) (TN/FP/FN/TP = 172/97/230/501; Figure 3H). Absolute performance on TN5000 was lower than on DDTI for all strategies, consistent with the higher malignancy prevalence and broader case mix.

For Gemini 2.5 Flash-Lite, all four strategies produced similar AUCs (0.619–0.658) with very high sensitivity but near-absent specificity (4.8–17.5%), indicating that the model classified almost all nodules as malignant (Table 5; Figure 3A–D). The hybrid strategy did not improve AUC over text prompting (ΔAUC +0.8%; *p* = 0.800).

### 3.4. Diagnostic Performance on the AUS Cohort

**Figure 4:**
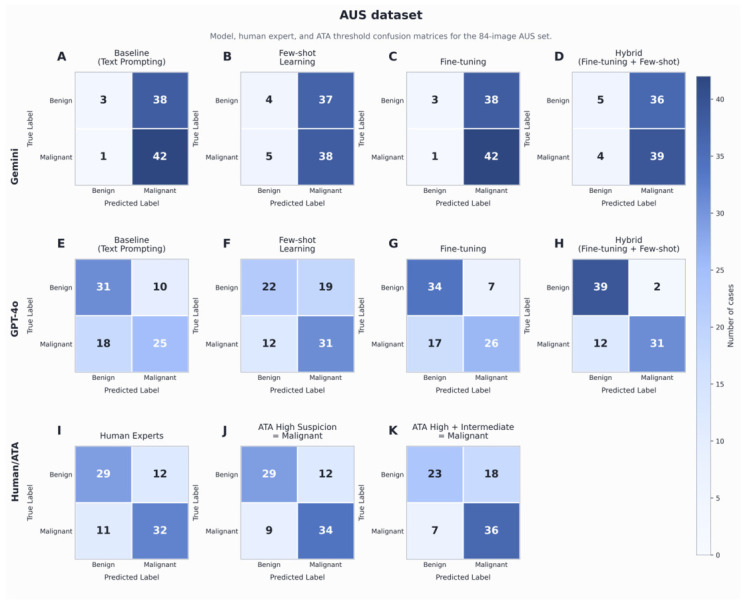
Confusion matrices on the AUS cohort and clinical benchmarks. Matrices are shown for Gemini 2.5 Flash-Lite (**A**–**D**) and GPT-4o (**E**–**H**) under each strategy: (**A**,**E**) text prompting, (**B**,**F**) few-shot learning, (**C**,**G**) fine-tuning, and (**D**,**H**) hybrid (fine-tuning + few-shot); (**I**) endocrinologist consensus; (**J**) ATA risk stratification with high suspicion = malignant; and (**K**) ATA risk stratification with high + intermediate = malignant. Cells show case counts; color intensity encodes count.

The performance of GPT-4o on the AUS cohort is detailed in Table 6, with the confusion matrices presented in Figure 4E–H. Text prompting achieved an AUC of 0.668 (95% CI: 0.563–0.768), sensitivity of 58.1%, and specificity of 75.6% (TN/FP/FN/TP = 31/10/18/25; Figure 4E).

Few-shot learning produced an AUC of 0.629 (95% CI: 0.523–0.734), which was not significantly different from text prompting (ΔAUC −5.8%; *p* = 0.481). Although sensitivity increased to 72.1%, specificity decreased to 53.7%, reflecting more false positives (TN/FP/FN/TP = 22/19/12/31; Figure 4F).

Fine-tuning improved the overall performance (AUC 0.717, 95% CI: 0.618–0.812), with a sensitivity of 60.5% and specificity of 82.9% (TN/FP/FN/TP = 34/7/17/26; Figure 4G), with fewer false positives than text prompting and few-shot learning.

Consistent with the DDTI findings, the hybrid strategy showed the strongest performance on the AUS cohort (AUC 0.836, 95% CI: 0.756–0.911), significantly outperforming text prompting (ΔAUC +25.1%; *p* < 0.001), few-shot learning (+32.9%; *p* < 0.001), and fine-tuning (+16.6%; *p* = 0.014). The hybrid model achieved a sensitivity of 72.1% and specificity of 95.1% (TN/FP/FN/TP = 39/2/12/31; Figure 4H), with an accuracy of 83.3%, precision of 93.9%, F1 of 0.816, and a pronounced reduction in false positives compared with all other strategies. Pairwise accuracy comparisons (mid-p McNemar test, against the hybrid strategy) confirmed the AUC-based ordering: On DDTI, the GPT-4o hybrid strategy was more accurate than text prompting (*p* = 0.003) and few-shot learning (*p* < 0.001); on the AUS cohort, the hybrid strategy was more accurate than text prompting (*p* < 0.001), few-shot learning (*p* < 0.001), and fine-tuning (*p* = 0.019).

For Gemini 2.5 Flash-Lite on the AUS cohort, AUCs were low across strategies (0.489–0.617) with very high sensitivity but minimal specificity (7.3–12.2%), and the hybrid strategy showed the lowest AUC (0.489; Table 7; Figure 4A–D). As for the other datasets, few-shot learning, fine-tuning, and the hybrid approach did not improve Gemini’s performance over text prompting.

### 3.5. Performance of Gemini 2.5 Flash-Lite Across Datasets

For Gemini 2.5 Flash-Lite, none of the optimization strategies improved discrimination over text prompting for any dataset (Table 3, Table 5 and Table 7; Figure 2, Figure 3 and Figure 4A–D). On the TN5000 test split, AUCs ranged from 0.619 to 0.658, and the performance of the hybrid strategy did not exceed that of text prompting (ΔAUC +0.8%; *p* = 0.800). On the AUS cohort, AUCs were low across strategies (0.489–0.617), with the hybrid strategy showing the lowest AUC (0.489), while on DDTI, text prompting gave the highest Gemini AUC (0.770), and the other strategies produced nonsignificant decreases. Across the higher-prevalence TN5000 and AUS cohorts, Gemini classified almost all nodules as malignant, producing very high sensitivity but near-absent specificity.

### 3.6. Comparison with Human Experts and Clinical Guidelines on the AUS Cohort

For clinical benchmarking, the performance of GPT-4o hybrid was compared with the consensus of three endocrinologists and the ATA risk stratification (Figure 1; Table 8). Human experts achieved an AUC of 0.722 (95% CI: 0.623–0.822), with a sensitivity of 74.4% and specificity of 70.7% (TN/FP/FN/TP = 29/12/11/32; Figure 4I). Under the primary threshold (high suspicion = malignant), ATA risk stratification achieved an AUC of 0.749 (95% CI: 0.655–0.842), sensitivity of 79.1%, and specificity of 70.7% (TN/FP/FN/TP = 29/12/9/34; Figure 4J). Under the less conservative threshold (high + intermediate = malignant), ATA sensitivity rose to 83.7% and specificity fell to 56.1% (TN/FP/FN/TP = 23/18/7/36; Figure 4K).

The hybrid model achieved an AUC of 0.836 (95% CI 0.756–0.911), which was not significantly different from the endocrinologist consensus (AUC 0.722; *p* = 0.072) or ATA risk stratification at the primary threshold (AUC 0.749; *p* = 0.123). The hybrid model showed higher specificity (95.1%) than the endocrinologist consensus (70.7%; *p* = 0.002) and ATA (70.7%; *p* = 0.001), with similar sensitivity (72.1% vs. 74.4% and 79.1%, respectively; *p* > 0.05). The specificity advantage persisted against the less conservative ATA threshold (95.1% vs. 56.1%; *p* < 0.001).

## 4. Discussion

Our study evaluated strategies to improve MLLMs for thyroid cancer detection on ultrasound, using a non-reasoning model, GPT-4o, and a reasoning model, Gemini 2.5 Flash-Lite. For GPT-4o, few-shot learning provided limited improvement in AUC compared with structured text prompting, whereas fine-tuning improved performance. Combining fine-tuning with few-shot learning yielded the most consistent gains across an external public dataset (DDTI), a higher malignancy-prevalence external test split (TN5000), and a clinically challenging AUS cohort with surgical pathology. By contrast, the same strategies yielded no benefit for Gemini 2.5 Flash-Lite. This incremental evaluation of four optimization strategies across two model families clarifies which approaches improve MLLM performance for thyroid cancer detection on ultrasound and shows that the effect of optimization depends on the underlying model.

Prior research showed that GPT-4o’s performance can be improved with few-shot learning [12,23,24]; however, our study found that few-shot learning did not improve diagnostic accuracy when added to a detailed ATA-anchored prompt. One plausible explanation for this is that the structured prompt already constrained the model’s reasoning, and a limited number of exemplars provided insufficient visual diversity to further refine feature recognition. Fine-tuning alone also provided only limited performance gains in our study. We noted the step-level loss curves suggest possible overfitting during GPT-4o fine-tuning, and combined with the small dataset for fine-tuning, may contribute to the result. Despite this, the hybrid strategy, in which few-shot exemplars were provided to the fine-tuned model at inference, outperformed fine-tuning alone and had the largest improvement. This pattern may reflect a corrective effect of the in-context exemplars, which directed the model toward representative benign and malignant sonographic patterns and may have partly counteracted a biased decision boundary learned from the small fine-tuning set. These findings suggest that fine-tuning and few-shot prompting may provide complementary rather than redundant information. We also noted that the hybrid strategy significantly improved AUC over fine-tuning alone in the AUS cohort and TN5000 test split dataset, whereas the smaller improvement observed in DDTI did not reach statistical significance. One possible explanation is that the fine-tuned model already performed well on the selected, relatively clear DDTI, leaving less room for few-shot exemplars to provide further benefit. In contrast, the lower fine-tuned baseline in the diagnostically ambiguous AUS cohort allowed a greater incremental benefit from exemplar-guided inference. The modest sample sizes may also have limited the statistical power to detect the smaller difference in DDTI. These explanations remain exploratory and require confirmation in larger cohorts.

The benefits of these optimization strategies did not extend to Gemini 2.5 Flash-Lite. Across all datasets, few-shot learning, fine-tuning, and the hybrid approach did not improve AUC relative to text prompting. In the higher malignancy-prevalence TN5000 and AUS cohorts, the model classified nearly all nodules as malignant, resulting in high sensitivity but very low specificity. The greater proportion of malignant cases during validation may have contributed to this prediction bias; however, the same pattern was not observed with GPT-4o, suggesting that an imbalance in validation alone does not fully explain the finding. Rather, the effects of optimization may depend on model-specific differences in pretraining, reasoning behavior, and fine-tuning implementation, which may influence how each model incorporates exemplars and task-specific data. We did not perform a systematic sweep of the reasoning budget, so we cannot exclude the possibility that a larger budget would alter Gemini’s performance; this remains a limitation and a target for future work. These findings caution against assuming that an optimization strategy effective for one model family will transfer directly to another and support model-specific validation before clinical use.

Our study also evaluated a clinically challenging scenario: thyroid nodules with AUS cytology. With an estimated malignancy risk of 15–30% in patients with AUS cytology, many patients undergo repeat biopsy or diagnostic surgery for benign disease. Previous reports describe the reduced performance of the ATA ultrasound risk stratification for AUS nodules [3]. In our cohort, the GPT-4o hybrid model showed significantly higher specificity than human experts and the ATA risk stratification. Because only image classification, instead of clinical management, biopsy avoidance, or surgical outcomes, was assessed, any effect on procedures remains hypothesis-generating and requires prospective evaluation.

Despite its high specificity, the GPT-4o hybrid model produced seven false negatives on DDTI and twelve on the AUS cohort. Because missed malignancies carry greater clinical cost than false positives, the model’s high-specificity profile is best suited to supporting the exclusion of malignancy in lower-risk presentations rather than serving as a stand-alone rule-in test. In practice, the model would function as an adjunct that prompts, rather than replaces, cytological or histological confirmation when clinical suspicion is present.

Deploying LLM outputs alongside clinicians introduces interaction risks, including overtrust and a “halo effect,” in which the apparent sophistication of model outputs leads the clinicians to have undue confidence in them, especially for borderline nodules [16]. Future studies should pair model outputs with calibrated uncertainty and evaluate response quality with validated structured instruments such as the Quality Analysis of Medical Artificial Intelligence (QAMAI) tool [25] or the Artificial Intelligence Performance Instrument (AIPI) [26] to reduce subjective or biased interpretation. Because performance can differ substantially across architectures, as our GPT-4o and Gemini comparison shows, future work should also compare optimization strategies across newer GPT versions and other multimodal models, such as Claude and Gemini, to distinguish model-specific from generalizable gains [17].

Our findings should be interpreted with consideration of several limitations. First, the AUS cohort was retrospective and from a single center; larger multicenter studies are needed to assess generalizability. Second, selecting a high-quality subset from DDTI may introduce selection bias toward more easily interpretable images, so performance in routine imaging with greater variability may be lower. Conversely, the higher-prevalence TN5000 test split showed lower absolute performance, reflecting the influence of case mix. Third, the strategies differ in terms of how data is used—no labeled images are used in text prompting, 10 in-context exemplars are used in few-shot learning, and 40 training images are used in fine-tuning—so the comparison reflects realistic deployment configurations under a fixed small development budget rather than a controlled equal-N comparison. This difference in image budgets is intrinsic to each strategy and may influence the fairness of direct comparisons. Fourth, we evaluated fixed model versions during a defined period (gpt-4o-2024-08-06 in November–December 2024, and Gemini 2.5 Flash-Lite). Because these models are updated rapidly and snapshots may be deprecated, the results have limited temporal generalizability and should be read as evidence that task-specific optimization improves a given model at a given time rather than as a fixed performance ceiling. Finally, because these models are accessed via commercial third-party APIs, clinical translation would require institutional data-governance review, de-identification pipelines, privacy and security compliance, and regulatory clearance; latency and per-image cost are additional constraints. We therefore position the optimized model as a decision-support adjunct for future prospective evaluation rather than a stand-alone diagnostic tool.

## 5. Conclusions

This study compared four optimization strategies—text prompting, few-shot learning, fine-tuning, and a hybrid strategy combining few-shot learning with fine-tuning—across two model families for ultrasound-based thyroid nodule malignancy classification. For the non-reasoning model, GPT-4o, the hybrid strategy achieved the best performance across the DDTI, TN5000, and AUS datasets, whereas it did not improve the performance of the reasoning model Gemini 2.5 Flash-Lite. In the diagnostically challenging AUS cohort, the optimized GPT-4o model achieved high specificity, suggesting a possible role in reducing false-positive classifications; its discriminative performance was also significantly better than that of ATA risk stratification when high- and intermediate-suspicion categories were considered positive for malignancy. However, its sensitivity of 72.1% means that roughly one in four malignant nodules was missed, so it cannot be used to rule out malignancy and would need to be paired with cytological or histological confirmation. This retrospective, single-center study included a small sample and did not assess prospective clinical outcomes; therefore, validation in larger, prospective multicenter cohorts, together with evaluation of workflow integration and governance safeguards, is required before clinical use can be considered.

## Figures and Tables

**Table 1:** Characteristics of datasets used for model development and evaluation.

Dataset	Intended Use	N (Total)	Malignant, *n* (%)	Benign, *n* (%)
DDTI ^a^	External evaluation	80	28 (35.0)	52 (65.0)
TN5000 ^b^	External evaluation (high prevalence)	1000	731 (73.1)	269 (26.9)
AUS ^c^	Clinical evaluation	84	43 (51.2)	41 (48.8)
PMC-OA ^d^	Fine-tuning & few-shot examples (development only)	60	26 (43.3)	34 (56.7)

^a^ DDTI: Digital Database of Thyroid Images. ^b^ TN5000: 1000-image test split of the TN5000 thyroid ultrasound dataset. ^c^ AUS: Atypia of Undetermined Significance. ^d^ PMC-OA: PubMed Central Open Access.

**Table 2:** Diagnostic performance of GPT-4o on the DDTI evaluation dataset.

Model/Strategy	AUC (95% CI)	Sens % (95% CI)	Spec % (95% CI)	Accuracy % (95% CI; *p* *)	Precision % (95% CI)	F1 (95% CI)	ΔAUC vs. Baseline	ΔAUC vs. Few-Shot	ΔAUC vs. Fine-Tuning
Text prompting	0.704 (0.594–0.807)	67.9 (50.0–84.4)	73.1 (60.8–84.6)	71.3 (60.5–80.0; *p* = 0.003)	57.6 (40.7–75.0)	0.623 (0.459–0.754)	—	—	—
Few-shot	0.647 (0.543–0.750)	42.9 (24.1–61.9)	86.5 (76.4–94.9)	71.3 (60.5–80.0; *p* < 0.001)	63.2 (39.1–84.2)	0.511 (0.308–0.667)	−8.1% (*p* = 0.281)	—	—
Fine-tuning	0.791 (0.690–0.885)	71.4 (53.8–87.9)	86.5 (76.6–95.6)	81.3 (71.3–88.3; *p* = 0.096)	74.1 (56.2–90.6)	0.727 (0.571–0.849)	+12.4% (*p* = 0.183)	+22.3% (*p* = 0.012)	—
Hybrid (fine-tuning + few-shot)	0.866 (0.774–0.946)	75.0 (56.7–90.6)	98.1 (93.8–99.8)	90.0 (81.5–94.8; ref)	95.5 (85.0–100)	0.840 (0.706–0.938)	+23.0% (*p* = 0.015)	+33.9% (*p* < 0.001)	+9.5% (*p* = 0.211)

AUC, area under the receiver operating characteristic curve; CI, confidence interval; Sens, sensitivity; Spec, specificity; F1, F1-score; Δ, difference; ref, reference. AUC 95% CIs from 1000-iteration bootstrap; sensitivity, specificity, and accuracy 95% CIs are Wilson intervals; precision and F1 95% CIs from 2000-iteration bootstrap. Precision = positive predictive value. ΔAUC is the relative change vs. the reference strategy. * Accuracy was compared against the hybrid strategy using the mid-p McNemar test; AUC differences were assessed with DeLong’s test. *n* = 80 (28 malignant, 52 benign). Malignant = positive class.

**Table 3:** Diagnostic performance of Gemini 2.5 Flash-Lite on the DDTI evaluation dataset.

Model/Strategy	AUC (95% CI)	Sens % (95% CI)	Spec % (95% CI)	Accuracy % (95% CI; *p* *)	Precision % (95% CI)	F1 (95% CI)	ΔAUC vs. Baseline	ΔAUC vs. Few-Shot	ΔAUC vs. Fine-Tuning
Text prompting	0.770 (0.659–0.869)	100.0 (87.9–100.0)	21.2 (12.2–34.0)	48.8 (38.1–59.5; *p* = 0.332)	40.6 (29.8–52.4)	0.577 (0.452–0.686)	—	—	—
Few-shot	0.702 (0.586–0.808)	100.0 (87.9–100.0)	28.8 (18.3–42.3)	53.8 (42.9–64.3; *p* = 1.000)	43.1 (31.8–55.2)	0.602 (0.476–0.708)	−8.8% (*p* = 0.278)	—	—
Fine-tuning	0.726 (0.615–0.829)	100.0 (87.9–100.0)	23.1 (13.7–36.1)	50.0 (39.3–60.7; *p* = 0.481)	41.2 (30.3–53.0)	0.583 (0.460–0.691)	−5.7% (*p* = 0.454)	+3.4% (*p* = 0.696)	—
Hybrid (fine-tuning + few-shot)	0.754 (0.643–0.852)	96.4 (82.3–99.4)	30.8 (19.9–44.3)	53.8 (42.9–64.3; ref)	42.9 (31.4–55.1)	0.593 (0.463–0.704)	−2.1% (*p* = 0.813)	+7.4% (*p* = 0.396)	+3.9% (*p* = 0.637)

AUC, area under the receiver operating characteristic curve; CI, confidence interval; Sens, sensitivity; Spec, specificity; F1, F1-score; Δ, difference; ref, reference. AUC 95% CIs from 1000-iteration bootstrap; sensitivity, specificity, and accuracy 95% CIs are Wilson intervals; precision and F1 95% CIs from 2000-iteration bootstrap. Precision = positive predictive value. ΔAUC is the relative change vs. the reference strategy. * Accuracy was compared against the hybrid strategy using the mid-p McNemar test; AUC differences were assessed with DeLong’s test. All Gemini arms used Gemini 2.5 Flash-Lite with thinkingBudget = 512. *n* = 80 (28 malignant, 52 benign).

**Table 4:** Diagnostic performance of GPT-4o on the TN5000 1000-image test split.

Model/Strategy	AUC (95% CI)	Sens % (95% CI)	Spec % (95% CI)	Accuracy % (95% CI; *p* *)	Precision % (95% CI)	F1 (95% CI)	ΔAUC vs. Baseline	ΔAUC vs. Few-Shot	ΔAUC vs. Fine-Tuning
Text prompting	0.573 (0.536–0.607)	28.5 (25.3–31.8)	89.2 (84.9–92.4)	44.8 (41.8–47.9; *p* < 0.001)	87.8 (82.9–91.3)	0.429 (0.390–0.468)	—	—	—
Few-shot	0.613 (0.574–0.651)	69.5 (66.1–72.7)	52.8 (46.8–58.7)	65.0 (62.0–67.9; *p* = 0.229)	80.0 (76.7–82.9)	0.744 (0.719–0.768)	+7.0% (*p* = 0.076)	—	—
Fine-tuning	0.615 (0.576–0.655)	74.1 (70.9–77.3)	48.0 (41.9–53.7)	67.1 (64.1–69.9; ref)	79.5 (76.2–82.3)	0.767 (0.743–0.793)	+7.3% (*p* = 0.092)	+0.3% (*p* = 0.955)	—
Hybrid (fine-tuning + few-shot)	0.689 (0.653–0.724)	68.5 (64.8–71.9)	63.9 (58.0–69.8)	67.3 (64.2–70.2; *p* = 0.798)	83.8 (80.3–86.5)	0.753 (0.726–0.778)	+20.2% (*p* < 0.001)	+12.4% (*p* < 0.001)	+12.0% (*p* < 0.001)

AUC, area under the receiver operating characteristic curve; CI, confidence interval; Sens, sensitivity; Spec, specificity; F1, F1-score; Δ, difference; ref, reference. AUC 95% CIs from 1000-iteration bootstrap; sensitivity, specificity, and accuracy 95% CIs are Wilson intervals; precision and F1 95% CIs from 2000-iteration bootstrap. Precision = positive predictive value. ΔAUC is the relative change vs. the reference strategy. * Accuracy was compared against the hybrid strategy using the mid-p McNemar test; AUC differences were assessed with DeLong’s test. *n* = 1000 (731 malignant, 269 benign).

**Table 5:** Diagnostic performance of Gemini 2.5 Flash-Lite on the TN5000 1000-image test split.

Model/Strategy	AUC (95% CI)	Sens % (95% CI)	Spec % (95% CI)	Accuracy % (95% CI; *p* *)	Precision % (95% CI)	F1 (95% CI)	ΔAUC vs. Baseline	ΔAUC vs. Few-Shot	ΔAUC vs. Fine-Tuning
Text prompting	0.653 (0.616–0.690)	98.1 (96.8–98.9)	4.8 (2.8–8.1)	73.0 (70.2–75.7; *p* = 0.009)	73.7 (70.8–76.4)	0.842 (0.822–0.860)	—	—	—
Few-shot	0.628 (0.591–0.667)	94.1 (92.2–95.6)	17.1 (13.1–22.1)	73.4 (70.5–76.0; *p* = 0.019)	75.5 (72.6–78.2)	0.838 (0.818–0.856)	−3.8% (*p* = 0.288)	—	—
Fine-tuning	0.619 (0.584–0.656)	98.8 (97.7–99.4)	5.2 (3.1–8.5)	73.6 (70.8–76.2; *p* = 0.050)	73.9 (71.1–76.6)	0.845 (0.826–0.863)	−5.2% (*p* = 0.075)	−1.4% (*p* = 0.695)	—
Hybrid (fine-tuning + few-shot)	0.658 (0.621–0.698)	96.6 (95.0–97.7)	17.5 (13.4–22.5)	75.3 (72.5–77.9; ref)	76.1 (73.2–78.7)	0.851 (0.832–0.870)	+0.8% (*p* = 0.800)	+4.8% (*p* = 0.146)	+6.3% (*p* = 0.072)

AUC, area under the receiver operating characteristic curve; CI, confidence interval; Sens, sensitivity; Spec, specificity; F1, F1-score; Δ, difference; ref, reference. AUC 95% CIs from 1000-iteration bootstrap; sensitivity, specificity, and accuracy 95% CIs are Wilson intervals; precision and F1 95% CIs from 2000-iteration bootstrap. Precision = positive predictive value. ΔAUC is the relative change vs. the reference strategy. * Accuracy was compared against the hybrid strategy using the mid-p McNemar test; AUC differences were assessed with DeLong’s test. All Gemini arms used Gemini 2.5 Flash-Lite with thinkingBudget = 512. *n* = 1000 (731 malignant, 269 benign).

**Table 6:** Diagnostic performance of GPT-4o on the AUS cohort.

Model/Strategy	AUC (95% CI)	Sens % (95% CI)	Spec % (95% CI)	Accuracy % (95% CI; *p* *)	Precision % (95% CI)	F1 (95% CI)	ΔAUC vs. Baseline	ΔAUC vs. Few-Shot	ΔAUC vs. Fine-Tuning
Text prompting	0.668 (0.563–0.768)	58.1 (42.5–73.2)	75.6 (61.1–88.6)	66.7 (56.1–75.8; *p* < 0.001)	71.4 (55.3–86.7)	0.641 (0.507–0.756)	—	—	—
Few-shot	0.629 (0.523–0.734)	72.1 (57.8–85.7)	53.7 (37.8–69.4)	63.1 (52.4–72.6; *p* < 0.001)	62.0 (47.9–75.0)	0.667 (0.548–0.772)	−5.8% (*p* = 0.481)	—	—
Fine-tuning	0.717 (0.618–0.812)	60.5 (45.0–75.6)	82.9 (70.0–94.3)	71.4 (61.0–80.0; *p* = 0.019)	78.8 (64.3–92.3)	0.684 (0.556–0.789)	+7.3% (*p* = 0.362)	+13.9% (*p* = 0.157)	—
Hybrid (fine-tuning + few-shot)	0.836 (0.756–0.911)	72.1 (57.9–85.4)	95.1 (87.2–99.0)	83.3 (73.9–89.8; ref)	93.9 (85.2–100)	0.816 (0.708–0.902)	+25.1% (*p* < 0.001)	+32.9% (*p* < 0.001)	+16.6% (*p* = 0.014)

AUC, area under the receiver operating characteristic curve; CI, confidence interval; Sens, sensitivity; Spec, specificity; F1, F1-score; Δ, difference; ref, reference. AUC 95% CIs from 1000-iteration bootstrap; sensitivity, specificity, and accuracy 95% CIs are Wilson intervals; precision and F1 95% CIs from 2000-iteration bootstrap. Precision = positive predictive value. ΔAUC is the relative change vs. the reference strategy. * Accuracy was compared against the hybrid strategy using the mid-p McNemar test; AUC differences were assessed with DeLong’s test. *n* = 84 (43 malignant, 41 benign). Malignant = positive class.

**Table 7:** Diagnostic performance of Gemini 2.5 Flash-Lite on the AUS cohort.

Model/Strategy	AUC (95% CI)	Sens % (95% CI)	Spec % (95% CI)	Accuracy % (95% CI; *p* *)	Precision % (95% CI)	F1 (95% CI)	ΔAUC vs. Baseline	ΔAUC vs. Few-Shot	ΔAUC vs. Fine-Tuning
Text prompting	0.617 (0.499–0.729)	97.7 (87.9–99.6)	7.3 (2.5–19.4)	53.6 (43.0–63.8; *p* = 0.754)	52.5 (41.7–63.1)	0.683 (0.579–0.773)	—	—	—
Few-shot	0.555 (0.429–0.677)	88.4 (75.5–94.9)	9.8 (3.9–22.5)	50.0 (39.5–60.5; *p* = 0.508)	50.7 (39.6–61.7)	0.644 (0.532–0.740)	−10.0% (*p* = 0.304)	—	—
Fine-tuning	0.567 (0.448–0.682)	97.7 (87.9–99.6)	7.3 (2.5–19.4)	53.6 (43.0–63.8; *p* = 0.754)	52.5 (41.7–63.1)	0.683 (0.584–0.773)	−8.1% (*p* = 0.368)	+2.2% (*p* = 0.818)	—
Hybrid (fine-tuning + few-shot)	0.489 (0.366–0.610)	90.7 (78.4–96.3)	12.2 (5.3–25.5)	52.4 (41.8–62.7; ref)	52.0 (40.9–62.9)	0.661 (0.559–0.754)	−20.7% (*p* = 0.040)	−11.9% (*p* = 0.315)	−13.8% (*p* = 0.141)

AUC, area under the receiver operating characteristic curve; CI, confidence interval; Sens, sensitivity; Spec, specificity; F1, F1-score; Δ, difference; ref, reference. AUC 95% CIs from 1000-iteration bootstrap; sensitivity, specificity, and accuracy 95% CIs are Wilson intervals; precision and F1 95% CIs from 2000-iteration bootstrap. Precision = positive predictive value. ΔAUC is the relative change vs. the reference strategy. * Accuracy was compared against the hybrid strategy using the mid-p McNemar test; AUC differences were assessed with DeLong’s test. All Gemini arms used Gemini 2.5 Flash-Lite with thinkingBudget = 512. *n* = 84 (43 malignant, 41 benign).

**Table 8:** Comparison of the GPT-4o hybrid model with human experts and clinical risk stratification on the AUS cohort.

Model/Reader Consensus	AUC (95% CI)	Sens % (95% CI)	Spec % (95% CI)	ΔAUC vs. Hybrid (*p*)	Δ Sens vs. Hybrid (*p*)	Δ Spec vs. Hybrid (*p*)
Hybrid (fine-tuning + few-shot)	0.836 (0.756–0.911)	72.1 (57.9–85.4)	95.1 (87.2–99.0)	—	—	—
Endocrinologist consensus	0.722 (0.623–0.822)	74.4 (60.5–87.2)	70.7 (55.6–84.4)	−13.6% (*p* = 0.072)	+3.2% (*p* = 0.999)	−25.6% (*p* = 0.002)
ATA—high suspicion = malignant	0.749 (0.655–0.842)	79.1 (64.0–90.0)	70.7 (54.5–83.9)	−10.3% (*p* = 0.123)	+9.7% (*p* = 0.607)	−25.6% (*p* = 0.001)
ATA—high + intermediate = malignant	0.699 (0.599–0.792)	83.7 (70.0–91.9)	56.1 (41.0–70.1)	−16.4% (*p* = 0.017)	+16.1% (*p* = 0.180)	−41.0% (*p* < 0.001)

AUS, atypia of undetermined significance; AUC, area under the receiver operating characteristic curve; CI, confidence interval; Sens, sensitivity; Spec, specificity; ATA, American Thyroid Association risk stratification system; Δ, difference. Differences (Δ) are expressed relative to the hybrid model. AUC differences were assessed with DeLong’s test; sensitivity and specificity differences with the mid-p McNemar test. Sensitivity and specificity 95% CIs are Wilson intervals.

## Data Availability

The data presented in this study are available on request from the corresponding author due to ethical reasons.

## Supplementary Materials

The following supporting information can be downloaded at https://www.mdpi.com/article/10.3390/diagnostics16121931/s1. Supplementary Materials: Prompt templates and inference workflow; Figure S1: Fine-tuning training and validation loss.

## Abbreviations

The following abbreviations are used in this manuscript:

ΔAUC	difference in area under the receiver operating characteristic curve
ATA	American Thyroid Association
AUC	area under the receiver operating characteristic curve
AUS	atypia of undetermined significance
CI	confidence interval
DDTI	Digital Database of Thyroid Images
FN	false negative
FNA	fine-needle aspiration
FP	false positive
MLLM	multimodal large language model
OR	odds ratio
PMC OA	PubMed Central Open Access
TIRADS	Thyroid Imaging Reporting and Data System
TN	true negative
TP	true positive
