# Supplementary material for: Optimizing a Multimodal Large Language Model for Ultrasound-Based Thyroid Nodule Malignancy Classification: A Comparative Study of Few-Shot Learning, Prompt Engineering, and Fine-Tuning
Source: Diagnostics (Basel). 2026 Jun 22;16(12):1931. doi: 10.3390/diagnostics16121931 (PMC13298059; doi:10.3390/diagnostics16121931)
Supplement: Supplementary file 1 [file diagnostics-16-01931-s001.zip › diagnostics-4359791-supplementary.pdf]

Supplementary Materials. Prompt Templates and Inference Workflow

This appendix provides representative prompt structures and an example interaction workflow for the four model configurations evaluated (baseline prompting, few-shot learning, fine-tuning, and hybrid). The examples below use placeholders and do not contain any patient-identifiable information.

S1. System prompt template

System message (template; applied to all configurations):
You are a clinician assisting with thyroid ultrasound interpretation. Analyze the provided thyroid ultrasound image and report the key ATA sonographic features:
1) Composition
2) Echogenicity
3) Shape (taller-than-wide vs wider-than-tall)
4) Margin
5) Echogenic foci

Then provide:
- A brief rationale referencing the observed features
- A final binary classification: Benign or Malignant
- Malignant (e.g., High, Intermediate, Low, Very Low Suspicion, or Benign)

S2. User message template

User message (template):
[Attach 1 thyroid ultrasound image]
Task: Classify the nodule as Benign or Malignant based on the ATA feature framework above. Do not use any external information beyond the image.

S3. Output structure (recommended)

To improve consistency, the prompt can request a structured output (free-text with headers or JSON). Example free-text structure:
Composition: ...
Echogenicity: ...
Shape: ...
Margin: ...
Echogenic foci: ...
Rationale: ...
Final classification (Benign/Malignant): ...
Confidence (optional): ...

S4. Few-shot exemplar formatting (few-shot and hybrid)

For few-shot learning, exemplars are inserted before the query image using the same structure as the requested output. Each exemplar consists of (i) an image, (ii) a concise feature description, and (iii) the ground-truth label. Example (placeholders):
Example 1 (Benign)
[Attach exemplar image 1]
Composition: predominantly cystic
Echogenicity: anechoic
Shape: wider-than-tall
Margin: smooth
Echogenic foci: none
Final classification: Benign

Example 2 (Malignant)
[Attach exemplar image 2]
Composition: solid
Echogenicity: hypoechoic
Shape: taller-than-wide
Margin: irregular
Echogenic foci: punctate echogenic foci
Final classification: Malignant

S5. Fine-tuning data format (fine-tuning and hybrid)

For fine-tuning, each training example pairs an image with an instruction and a reference response. A JSONL example schema is shown below:

```
{
  "messages": [
    {"role": "system", "content": "...system prompt template..."},
    {"role": "user", "content": [
      {"type": "input_text", "text": "Classify the thyroid nodule as Benign or Malignant using ATA features."},
      {"type": "input_image", "image_url": "<image_or_file_reference>"}
    ]},
    {"role": "assistant", "content": "Composition: ...\n...\nFinal classification: Malignant"}
  ]
}
```

S6. Fine-tuning training and validation loss

Supplementary Figure S1 shows the fine-tuning training and validation loss for both models. For GPT-4o, the OpenAI interface exposes step-level training loss and periodic validation loss; for Gemini 2.5 Flash-Lite, Vertex TensorBoard scalar metrics are shown.

Supplementary Figure S1.

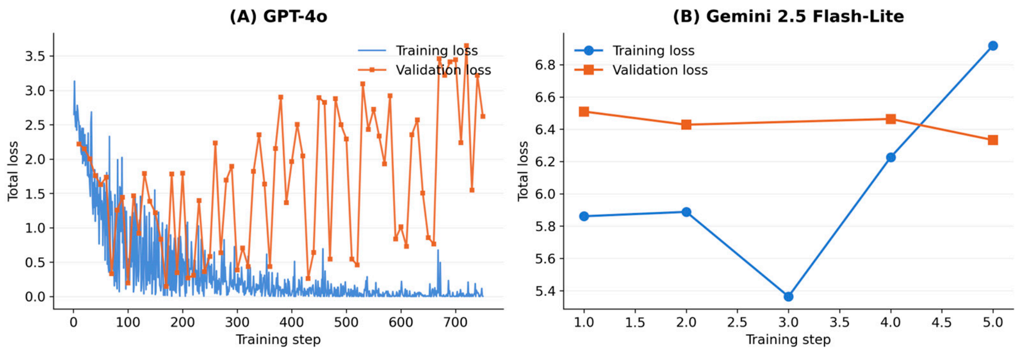

Figure S1. Fine-tuning training and validation loss. (A) GPT-4o (gpt-4o-2024-08-06) fine-tuning job: step-level training loss decreased toward zero while periodic validation loss remained high and variable, consistent with overfitting on the small (40-image) training set (final training loss ≈ 0.003; final validation loss ≈ 2.62); the default learning-rate multiplier (1.0), 3 epochs, and batch size 1 were retained to limit this overfitting. (B) Gemini 2.5 Flash-Lite supervised tuning (base model gemini-2.5-flash-lite; 3 epochs; learning-rate multiplier 0.5; adapter size ADAPTER_SIZE_ONE; 40 training images, 10 validation images): training and validation loss across the five logged steps (final training total loss ≈ 6.92; final validation total loss ≈ 6.33, with no decrease over tuning, indicating that supervised tuning did not converge to an improved fit). Training loss, blue; validation loss, orange.
